# Normative Data for the Famous People Fluency Test in the Adult French-Quebec Population and Validation Study in Mild Cognitive Impairment and Alzheimer’s Disease

**DOI:** 10.1093/arclin/acae053

**Published:** 2024-07-14

**Authors:** Joël Macoir, Mariane Landry, Carol Hudon

**Affiliations:** Faculté de médecine, École des Sciences de la réadaptation, Université Laval, Québec QC, Canada; Centre de recherche CERVO – Brain Research Centre, Québec QC, Canada; Centre de recherche CERVO – Brain Research Centre, Québec QC, Canada; Faculté des sciences sociales, École de psychologie, Université Laval, Québec QC, Canada; Centre de recherche VITAM, Québec QC, Canada; Centre de recherche CERVO – Brain Research Centre, Québec QC, Canada; Faculté des sciences sociales, École de psychologie, Université Laval, Québec QC, Canada; Centre de recherche VITAM, Québec QC, Canada

**Keywords:** Verbal fluency, Assessment, Norms/normative studies, Test validity

## Abstract

**Objective:**

The production of words in verbal fluency tests relies heavily on executive functions and linguistic abilities. New tests such as the famous people fluency test can also be useful in clinical practice and research. This test, in which participants are asked to name so many famous people, has the potential to distinguish healthy individuals from participants with neurological disorders such as mild cognitive impairment or Alzheimer’s disease.

**Method:**

The aim of this study was to determine the psychometric validity of the test (Study 1) and to provide normative data in the adult population of French Quebec for the famous people fluency test (Study 2).

**Results:**

The results of the normative study, derived from a sample of 378 healthy individuals between the ages of 50 and 92, showed that age and educational level significantly influence performance on the test. Therefore, percentile ranks were calculated for performance on the famous people fluency test, stratified for these two variables. The results of Study 2 showed that the test differentiated the performance of healthy participants from the performance of participants with mild cognitive impairment or Alzheimer’s disease. The results also showed that the famous people fluency test has adequate convergent validity, established with a semantic fluency test, and that the results showed good stability over time (test–retest validity).

**Conclusion:**

Norms and psychometric data for the famous people fluency test will improve the ability of clinicians and researchers to better recognize executive and language impairments associated with pathological conditions.

## INTRODUCTION

Verbal fluency tests serve as essential tools in both clinical practice and research. They are mainly used to assess executive functions as they recruit mental flexibility, inhibition, short-term memory, and information updating (Amunts et al., 2021). The score in verbal fluency tests is based on the number of words produced by the examinee, so that language processes such as activation of semantic knowledge, lexical retrieval, and attention are also addressed (Pekkala, 2012; Whiteside et al., 2016). The two most used tests of verbal fluency are: (a) phonemic verbal fluency, in which participants are asked to generate words beginning with a specific letter within a limited time frame, and (b) semantic verbal fluency, in which participants are required to generate words belonging to a specific semantic category within a given time constraint (Troyer, 2000; Villalobos et al., 2023).

However, there are other forms of verbal fluency tests that have proven useful in differentiating the performance of healthy people from that of patients with brain damage. This is the case for the example of verb fluency (Macoir & Hudon, 2023a; Piatt et al., 2004), alternating verbal fluency (Ellfolk et al., 2014; Macoir & Hudon, 2023b), and orthographic constraint fluency (Macoir & Hudon, 2023b). A few studies have also used famous people verbal fluency tests as a measure of cognitive function. These tests typically involve asking participants to name as many famous people as they can within a specified time limit. We found two studies in which healthy adult participants were asked to produce as many names of famous people (Özdemir & Tunçer, 2021) or names of French and American presidents (Piolino et al., 2007) as possible. In pathological populations, we found two articles conducted with 21 participants with mild cognitive impairment (MCI) (Gardini et al., 2015) and in two patients with post-stroke aphasia (Martins & Farrajota, 2007). In the study by Gardini and colleagues (2015), participants with MCI produced significantly fewer items than healthy older controls in various semantic categories, including politicians and singers. In 2007, Martins and Farrajota described two individuals with post-stroke aphasia who performed differently on experimental tasks exploring semantic and lexical processing, including phonemic, semantic, and famous people verbal fluency tasks. ACB, the patient with an ischemic lesion of the left hemisphere affecting the temporal lobe including the temporal pole, had significantly lower scores on the semantic and the phonemic fluency tasks, whereas he was at control level on the famous people fluency task. An opposite pattern was found in JFJ, the other patient, in whom the lesion affected the left hippocampus and the inferior temporo-occipital region, whereas the left temporal pole was spared. In a recent study (Macoir et al., 2024), we showed that participants with subjective cognitive decline produced significantly fewer famous names in a famous people fluency task than healthy participants. These results suggest that retrieving the names of famous people may be more difficult for people with aphasia, MCI, and even people with cognitive complaints.

There is a particular interest in exploring the activation of semantic representations of unique entities in neuropsychological assessment. Compared to object concepts such as animals or tools, unique entities are encoded in semantic memory at a very specific conceptual level (Grabowski et al., 2001). Semantic knowledge of object concepts is characterized by multimodal features (e.g., auditory, visual, encyclopedic) that are shared by different concepts and allow generalization across different instances and modalities (Rogers et al., 2006). In contrast, unique entities such as famous people are associated with very specific and little shared semantic knowledge (e.g., Céline Dion is a famous singer born in the province of Quebec), are also often associated with episodic representations (e.g., I remember a performance of Céline Dion in Montreal) and are referred to by proper names (Wang et al., 2016). According to some theoretical models of semantic memory, such as the Hub account (Rogers et al., 2006), the two types of concepts are neuroanatomically supported by the anterior temporal lobes. However, these two lobes are more heavily engaged when the task requires the discrimination of very similar concepts at a specific level, such as unique entities, compared to more general concepts that share many properties, such as object concepts (Patterson et al., 2007; Tyler et al., 2004).

These differences between object concepts and unique entities are also found in dissociations reported in clinical studies, particularly those examining semantic processing in normal and pathological aging. For example, Haslam and colleagues (2004) found that healthy participants aged 50–65 were less accurate than participants aged 20–35 in providing semantic information about famous people. As an example from neuropsychology, Joubert and colleagues (2008) found that knowledge of famous people and famous events is more impaired than knowledge of objects in people with MCI. As for the studies in famous people verbal fluency, this pattern of performance in healthy and brain-damaged people suggests that unique entities are more vulnerable than object concepts and are therefore more prone to semantic breakdown following neurological impairment. The cognitive processes involved in the retrieval of semantic information about famous people in the studies by Haslam and colleagues (2004) and Joubert and colleagues (2008) and the generation of names of famous people in a fluency task are not equivalent. Both tasks recruit the activation of semantic knowledge in semantic memory, but the famous people fluency task also involves the activation of phonological lexical forms as well as executive functions, as in all other types of verbal fluency. This justifies the relevance of using the famous people verbal fluency task in clinical and research settings as a complement to neuropsychological instruments.

The first objective (Study 1) was to determine the known-group validity, the convergent validity, and the test–retest validity of the famous people fluency test. To the best of our knowledge, there are no normative data for the famous people verbal fluency test. The second objective of this cross-sectional study was to examine the relationship between performance on this test and sociodemographic variables such as age, education, and sex in order to provide normative data for individuals living in the French-speaking community of Quebec, Canada (Study 2).

### Study 1. Known-Group Validity, Convergent Validity and Test–Retest Reliability of the Famous People Fluency Test

The aim of Study 1 was to determine the known-group validity, convergent validity, and test–retest validity of the famous people fluency test.

## METHODS

### Known-Group Validity

To establish known-group validity, we tested whether scores on the famous people fluency test differed between the performance of healthy controls (HCs), participants with MCI, and participants with AD. Based on the impairment of semantic processing, lexical access, and executive dysfunction (Callahan et al., 2015; de Mendonça et al., 2021; Kirova et al., 2015; Taler & Phillips, 2008), we hypothesized that the performance of participants with MCI and AD would be significantly lower than that of HCs. The description of the sociodemographic characteristics of the participants in the three groups can be found in Table 5.

#### Participants

The MCI group comprised 19 participants diagnosed with MCI according to the clinical criteria outlined by Albert and colleagues (2011): (a) cognitive concerns reflecting a change in cognition reported by the patient, an informant, or the clinician (i.e., historical or observed evidence of decline over time); (b) objective evidence of impairment (greater than −1.5 *SD* based on normative data for age, sex, and education) in one or more cognitive domains, including episodic memory; (c) no significant impairment in functional abilities based on clinical consensus and the Alzheimer's Disease Cooperative Study-Activities of Daily Living (ADCS-ADL); and (d) not demented.

The AD group comprised 22 individuals diagnosed with probable AD, as per the current diagnostic criteria (McKhann et al., 2011), confirmed through patients’ medical records and history, such as a diagnosis by a medical doctor and/or receiving approved pharmacological dementia treatment. The included participants with probable AD were at the mild stage of the disease.

The HC group comprised 19 participants from Study 2 (normative data), who were selected to match the AD and MCI participants on demographic characteristics to avoid bias in these variables. They exhibited good physical and mental health, reported no noteworthy subjective cognitive complaints, and demonstrated general cognitive performance within the normal range on the Montreal Cognitive Assessment (MoCA) test (Nasreddine et al., 2005), based on French-Quebec normative data (Larouche et al., 2016).

The exclusion criteria for all participants were: (a) a history of moderate or severe traumatic brain injury, (b) a history of cerebrovascular disease, (c) a history of delirium within the past 6 months, (d) a history of intracranial surgery, (e) a history of neurological disorders of cerebral origin not investigated in the study, (f) a history of encephalitis or bacterial meningitis, (g) unstable metabolic or medical conditions (e.g., untreated hypothyroidism or diabetes), (h) an active and unstable psychiatric syndrome, (i) alcoholism or substance abuse, (j) uncorrected vision or hearing impairments, (k) a cancer treatment in the past 12 months; (l) a general anesthesia in the past 6 months; (m) a history of psychotic symptoms or manic episodes, and (n) inability of the participant to provide consent due to inaptitude.

#### Statistical analyses

Chi-square analysis was conducted to test for differences in sex distribution across the three groups. A Kruskal–Wallis test was used to compare the three groups in terms of their level of education, while analyses of variance (ANOVAs) were used to compare their age and performance on the famous people fluency test. Pairwise comparisons were computed using paired *t*-tests with a Bonferroni correction. Effect sizes were expressed as partial Eta squared (η_p_^2^) and interpreted according to Cohen (1988), i.e., values in the range of 0.01–0.06 indicate a small effect of the main factor, whereas Eta squared values in the range of 0.06–0.14 and above 0.14 indicate moderate and strong effects, respectively. All statistical analyses were performed using Jamovi 2.5 (The Jamovi Project, 2024), with the alpha level set at 0.05.

#### Results

As shown in Table 5, the three groups were statistically equivalent in terms of sex (*χ^2^* = 0.17, *df* = 2, *p* = .92). The three groups were also statistically equivalent in terms of age, *F*(2, 79) = 0.059, *p* = .94, and education level *χ^2^* = 0.70, *df* = 2, *p* = .705. As expected, the three groups were statistically different with respect to general cognition measured with the MoCA, *F*(2, 79) = 45.2, *p* < .001. Post-hoc tests revealed that: (a) the performance of HCs differed significantly from participants with MCI (*p* < .001) and participants with AD (*p* < .001); b) the performance of participants with MCI differed significantly from participants with AD (*p* < .01).

With respect to the famous people fluency test, the ANOVA revealed a main effect of group for the total score, *F*(2, 79) = 12.3, *p* < .001. Post-hoc tests revealed that the performance of HCs differed significantly from participants with MCI (*p* = .01) and participants with AD (*p* < .001), whereas the performance of participants with MCI did not significantly differ from participants with AD (*p* = .48).

### Convergent Validity

Convergent validity was established through the administration to HCs of the famous people fluency test and a semantic fluency task in which participants were asked to generate as many vegetable names as possible in 90 s. As both tests involve the cognitive processes of semantic activation, lexical access, and executive functions, a positive correlation was expected.

#### Participants

The two tests were administered to the 99 healthy (46 men, 53 women) participants (mean age = 66.80, *SD* = 6.47 years; mean education = 17.2, *SD* = 3.24 years; mean MoCA score = 27.0, *SD* = 2.12), of the normative study (see Study 2) who also completed the semantic verbal fluency test.

#### Statistical analyses

The relationship between the participants’ performance on the two tests was analyzed using the Pearson correlation coefficient.

#### Results

As expected, the famous people fluency test (mean number of famous names = 14.9, *SD* = 4.49) correlated significantly and positively (*r* = .335, *p* < .001) with the semantic verbal fluency task (mean number of vegetables = 16.8, *SD* = 5.42) suggesting that the two tests assess the same cognitive construct.

### Test–Retest Reliability

The famous people fluency test was administered twice to a group of healthy participants. There are a variety of administration intervals in the literature that are used in test–retest studies. Since repeated administrations of a verbal fluency task can influence performance in healthy individuals due to a learning effect (Lemay et al., 2004), a long interval of 3 months was chosen in this study (mean = 88.1 days).

#### Participants

This procedure was administered to a group of 22 healthy participants (2 men; 20 women) participants (mean age = 67.80, *SD* = 9.35 years; mean education = 15.1, *SD* = 3.43 years; mean MoCA score = 27.0, *SD* = 1.85), who also participated in the normative study (see Study 2) and agreed to be assessed twice with the same test.

#### Statistical analyses

The two measures (T1 and T2) were compared using Bland–Altman analysis (Bland & Altman, 1986) and the intraclass correlation coefficient (ICC).

#### Results

Bland–Altman analysis showed that the limits of agreement at 95% of difference values on the famous people fluency test ranged from −8.4 [95% CI −11.6, −5.1] to 8.2 [95% CI 4.9, 11.4]. All results fell within the 95% CI. The “bias” or average difference line was close to zero (−0.09 [95% CI −1.96, 1.78]), and the relative symmetry in the distribution of points above and below this line indicated that participants performed similarly on the two measures. In fact, the performance of the healthy participants did not differ statistically between T1 and T2 (T1 mean = 14.0, *SD* = 6.19; T2 mean = 14.0, *SD* = 5.58; ICC = 0.754, *p* < .001), indicating that the scores showed good stability over time.

### Study 2. Normative Data

The aim of Study 2 was to develop normative data for the famous people fluency test adapted to adults and elderly from Quebec.

## METHODS

### Participants

Speech-language pathology students and research assistants in the province of Quebec (Canada) recruited healthy adults living in the community through public advertisements and referrals from their acquaintances. French was the mother tongue and main language for all of them, defined as the first language learnt at home in childhood and that the person still spoke at the time of data collection (Statistique Canada, 2022). Participants were excluded if they met one (or more) of the following criteria that may affect cognition: (a) a z-score of −1.33 or less on the MoCA (Nasreddine et al., 2005) according to research team standards (Larouche et al., 2016); (b) a subjective cognitive complaint as in people with subjective cognitive decline (Jessen et al., 2020); (c) an active and unstable psychiatric syndrome; (d) a history of moderate or severe traumatic brain injury; (e) a history of stroke; (f) a history of intracranial surgery; (g) a neurological disease; (h) an untreated medical or metabolic disease; (i) a delirium in the past 6 months; (j) an electroconvulsive therapy in the past 12 months; (k) a cancer treatment in the past 12 months; (l) a general anesthesia in the past 6 months; (m) a history of psychotic symptoms or manic episodes; (n) a history of substance abuse; (o) medications that may impair language and memory function (e.g., anticholinergic medications); and (p) uncorrected hearing problems. This exclusion criteria information was obtained from participant self-reports.

Participants’ ages ranged from 50 to 92 years, with education levels spanning from 7 to 23 years. Educational attainment was treated as a continuous variable, but for clarity, it was contextualized within the Quebec education system: elementary school corresponds to 6 years; high school diploma, 11 years; college diploma, 13 years (14 for technical degrees); bachelor's degree, 16 years; master's degree, 18 years; doctoral degree, 21 years; and medical or postdoctoral fellowship, 23 years. When participants attained multiple degrees, the highest level was considered. Duplicate degrees at the same level (e.g., two master’s degrees) were recorded at the master’s level only (i.e., equivalent degrees were not combined or added). Special cases were addressed individually, such as professional education with or without a high school diploma.

The study (Project No. 2019-1541) was approved by the Comité d'Éthique de la Recherche Sectoriel en Neurosciences et Santé Mentale [Ethics Committee for Sectorial Research in Neurosciences and Mental Health], and all participants gave written informed consent to participate.

### Material and Procedure

Participants were given the following instructions orally (in French), “I want you to name as many famous people as possible, e.g., the names of singers, athletes, politicians, actors. It must be a person who already existed, not a fictional character. You must tell me the first and last name of the famous person, not just the first or last name. Can you give me an example of the name of a famous person?” If the answer is inappropriate, the examiner asks for another example of a famous person. If the answer is acceptable, the examiner says, “Great. Before you begin, do you have any questions? To avoid distraction, you must now close your eyes and tell me as many names of famous people as possible in 1 minute 30.” At the end of the task, if the participant has given names unknown to the examiner, the examiner says: “You said “FIRST NAME + LAST NAME”. I do not know this person. Can you tell me why s/he is famous?”

The scoring method was based on the number of different names of famous people produced in 90 s. Responses that referred to famous people whose last names were not commonly used, were mostly unknown (e.g., Queen Elizabeth, Napoleon), or did not exist (e.g., “French singer” known only by her nickname “Zaz”) were also scored one point. When the person’s name evoked by the participants was unknown to the test administrators, the test administrators were instructed to search the Internet to validate the response. The criteria for the final decision that the person is/was famous were: belonging to a well-known field of notoriety (e.g., singers and musicians, athletes, politicians, actors, TV presenters or animators) and the existence of information about the person on the Internet.

This study is part of a broader validation and normative study that includes several cognitive and language assessments. Participants attended two 90-min assessment sessions at XXX. In the first session, they gave written informed consent and completed a sociodemographic questionnaire. Then, the MoCA was administered to confirm cognitive health. During this session, participants also underwent the famous people verbal fluency test, the Mini-SEA (Mulet-Perreault et al., 2024) and psychomotor speed tests (Grooved Pegboard Test; Lafayette Instrument, 2023), visual episodic memory (Location Learning Test; Bucks & Willison, 1997), inhibition (Hayling’s Test; Burgess & Shallice, 1997) and language (naming action verbs—DVAQ-30 Test; Macoir et al., 2023). The tests were administered in a predetermined order to avoid potential interference. Additional protocol tests were administered in the second session.

Trained and supervised research assistants conducted assessments of participants. Evaluations occurred individually, either in a serene environment at the participants’ homes or in a designated quiet space at the research center. Participants were provided uninterrupted time during tasks, with instructions reiterated as needed if they became disoriented or forgot their tasks. Responses were meticulously recorded word-for-word. Recruitment and assessments were carried out from June 2017 to May 2022.

### Statistical Analyses

The total score was selected as the variable of interest for normalization. First, the data distributions of the normative sample were checked for skewness using the Shapiro–Wilk test and histograms. All data distributions were positively skewed, and no transformation was possible to normalize the distribution of scores. Therefore, partial Spearman rank correlations were calculated to assess the relationship between the sociodemographic characteristics of the participants (age and educational level) and the total score on the fluency test. The influence of sex on the fluency scores was analyzed using the Mann–Whitney U test.

As skewness does not affect percentiles, they offer valuable insights into the typical or atypical occurrence of a test score within the normative population (Crawford & Garthwaite, 2009). Consequently, percentile ranks were computed and categorized according to significant correlations between sociodemographic factors and scores.

All statistical analyses were performed using Jamovi 2.5 (The jamovi project, 2024), with the alpha level set at 0.05.

## RESULTS

The initial normative sample consisted of 392 participants living in the community, aged between 50 and 92 years and with a formal education comprised between 7 and 23 years. A total of 14 of them were withdrawn because of aberrant scores (i.e., scores below 1.33 *SD* from the mean) in the MoCA (Nasreddine et al., 2005). Thirty-two of them took part in an earlier study involving people with subjective cognitive decline (Macoir et al., 2024). The final normative sample comprised 378 individuals living in the community, consisting of 238 females and 140 males, aged between 50 and 92 years (mean age = 67.0 years; *SD* = 8.8). Their formal education spanned from 7 to 23 years (mean education level = 15.4 years; *SD* = 3.28). All participants demonstrated normal cognition, as evidenced by their normal age-, sex-, and education-adjusted scores (Larouche et al., 2016) on the MoCA, with a mean score of 27.1 (*SD* = 1.83). Detailed characteristics of the participants, including age, sex, and educational level, are presented in Table 1.

**Table 1:** Comparison of HC, MCI, and AD participants on sociodemographic variables and fluency tests

Characteristics	HC (*n* = 41)	MCI (*n* = 19)	AD (*n* = 22)	*p*	Effect size (*n_p_^2^*)
Age, mean (*SD*)	75.0 (6.62)	75.5 (6.19)	74.8 (6.81)	.943	.001
Education, mean (*SD*)	14.9 (3.27)	14.1 (3.15)	14.5 (3.91)	.705	-
Female, *n* (%)	18 (43.90)	9 (47.37)	9 (40.91)	.92	-
MoCA, mean (*SD*)	26.1 (2.16)	23.1 (2.71)	19.2 (3.66)	<.001^a^^,^^b^^,^^c^	.533
Famous people fluency total score	13.1 (4.45)	9.5 (5.09)	7.55 (3.81)	<.001^a^^,^^b^	.237

*Note*s. Effect size: n_p_^2^ from 0.01 to 0.06 = small effect; n_p_^2^ from 0.06 to 0.14 = moderate effect; n_p_^2^ higher than .14 = strong effect. HC = healthy controls; MCI = Mild Cognitive Impairment; AD = Alzheimer’s Disease

^a^Symbols for significant post hoc group differences: HC versus MCI.

^b^Symbols for significant post hoc group differences: HC versus AD.

^c^Symbols for significant post hoc group differences: MCI versus AD.

The participants were distributed across different age groups. However, compared to the demographic data from Quebec, women and people with a higher level of education were overrepresented. A comparison between the demographic data from the province of Quebec (Institut de la statistique du Québec, 2016, 2021) and the current study is illustrated in Table 2.

**Table 2:** Distribution of participants in the normative sample

**Characteristics**	***n***	**%**
**Age (years)** 50–55 56–60 61–65 66–70 71–75 76–80 81–85 85–92	46 41 85 71 72 38 17 8	12.17 10.85 22.49 18.78 19.05 10.05 4.50 2.12
**Women**	238	62.96
**Education (years)** 7–10 11–15 16+	16 162 200	4.23 42.86 52.91
**Cognitive screening** MoCA score	**Mean (*SD*)** 27.1 (1.83)	

Table 3 shows the descriptive statistics of the participants for the total score in the famous people fluency test.

**Table 3:** Comparison of demographic data between the province of Quebec (%) and the study sample (number and %)

**A. Sex distribution**				
	Province of Quebec		Study sample (*n* = 378)	
	*Males*	*Females*	*Males (n = 140, 37.04%)*	*Females (n = 238, 62.96%)*
50–59 years 60–69 years	50.4% 49.5%	49.6% 50.5%	26 (34.2%) 50 (33.6%)	50 (65.8%) 99 (66.4%)
70–79 years 80+ years	48.0% 40.1%	52.0% 59.9%	48 (42.1%) 16 (41.0%)	66 (57.9%) 23 (59.0%)
**B. Distribution by age and level of schooling (years of education)**				
	***0–12***	***13+***	***0–12 (n = 86, 22.75%)***	***13+ (n = 292, 77.25%)***
45–54 years*	52.7%	47.3%	8 (21.6%)	29 (78.4%)
55–64 years	61.9%	38.1%	20 (18.7%)	87 (81.3%)
65–74 years	65.2%	34.8%	32 (19.75%)	130 (80.25%)
75+ years	77.5%	22.5%	26 (36.1%)	46 (63.9%)

^*^For the study sample, 45–54 years

Spearman correlations showed that age was negatively correlated with the total score of the famous people fluency test (*r* = −.030, *p* < .001), indicating that performance decreases with age. The level of education correlated positively with the total score of the famous people fluency test (*r* = .17, *p* = < .001), meaning that performance increases as education increases. The Mann–Whitney U test, with sex as the independent variable, showed no significant effect of sex in the famous people fluency test (*U* = 15,529, *p* = .269), indicating that women and men performed at a similar level.

The 1st, 2nd, 5th, 8th 10th, 15th, 25th, 35th, 45th, 50th, 55th 65th, 75th, 85th, 90th, 95th, 98th, and 99th percentiles for the total number of responses in the famous people fluency test were calculated as a function of age and level of education. After visual inspection of the box-and-whisker plots, the 8th percentile was selected as the most reliable cutoff value as recommended by Guilmette and colleagues (2020) in their consensus statement of the American Academy of Clinical Neuropsychology. A score equal to or below the suggested cutoff score should be considered to fall below normal performance limits. Table 4 shows the descriptive statistics for the normative data as a function of age and educational level. When the number of famous people names corresponding to the cutoff score is similar to that of percentiles 10 and 15 (50–69 years, **≤**12 years of education) or percentile 10 (50–69 years, 13+ years of education), the clinician must use the score corresponding to percentile 5 to identify performance below the normal range.

**Table 4:** Descriptive statistics of participants on the famous people fluency test

	1st time interval score (0–30 s)	2nd time interval score (31–60 s)	3rd time interval score (61–90 s)	Total score
*M*	7.01	4.14	3.62	14.8
*SD*	2.46	2.16	2.22	5.02
*Min*	2	0	0	6
*Max*	17	14	11	37

**Table 5:** Descriptive statistics of the four groups of participants for the normative study as a function of age and education

	Age (years)			
	50–69 (*n* = 225)		70+ (*n* = 153)	
	Level of education			
	≤12 years (*n* = 44)	13+ years (*n* = 181)	≤12 years (*n* = 42)	13+ years (*n* = 111)
Age	60.80 (5.68)	61.2 (5.22)	76.8 (5.36)	75.1 (4.84)
Education	11.2 (0.795)	16.7 (2.46)	10.8 (1.35)	16.8 (2.50)
MoCA score	27.0 (1.76)	27.6 (1.57)	25.9 (2.25)	26.9 (1.82)
Total fluency score: mean, (*SD*), range	14.8 (4.68) 8–26	16.1 (5.29) 7–37	12.2 (3.71) 6–19	13.5 (4.42) 6–29
Percentiles				
1	8	8	6	6
2	8	8	7	7
5	8	9	7	8
8 (cutoff)	9	10	7	8
10	9	10	8	9
15	9	11	8	9
25	11	12	9	10
35	12	13	10	11
45	13	15	11	12
50	14	15	13	13
55	15	16	13	14
65	16	18	14	15
75	18	19	16	16
85	19	21	16	19
90	22	23	17	20
95	23	25	18	21
98	24	29	18	23
99	25	31	19	23
Total *N*	378			

## DISCUSSION

Verbal fluency tests stand as among the most frequently used tests in clinical practice and research (Amunts et al., 2020). They are characterized by quick and uncomplicated administration and scoring, require no special equipment, can differentiate between healthy individuals and those with neurological disorders, and are highly sensitive to cognitive decline (e.g., Olmos-Villaseñor et al., 2023; St-Hilaire et al., 2016). Phonemic and semantic tests of verbal fluency are the most used tests of verbal fluency. Apart from the traditional phonemic and semantic tests of verbal fluency, various less conventional forms of verbal fluency tests exist. These include alternating fluency (Downes et al., 1993; Macoir & Hudon, 2023b), orthographic constraint fluency (Macoir & Hudon, 2023b), and verb fluency (Macoir & Hudon, 2023a; Piatt et al., 1999).

The famous people fluency test used in this study requires different cognitive abilities. As with the semantic verbal fluency tests, activation of the semantic and lexical representations of famous people in the semantic and lexical long-term memory is just as necessary as attentional control of the task through the executive functions of mental flexibility, inhibition, strategic search, and short-term memory (Aita et al., 2018; Shao et al., 2014). Whereas concrete concepts represented by common names comprise a collection of properties shared by different entities associated with the same concept, unique concepts such as famous people or famous buildings are characterized by their distinctiveness and correspond to individual concepts and specific names. Unique concepts were found to be more vulnerable to neurological disorders compared to concrete concepts such as objects or actions (Ross & Olson, 2012; Thompson et al., 2002). For example, studies have shown impairment in naming pictures of famous people in individuals with MCI or AD (Ahmed et al., 2008; Clague et al., 2011; Estevez-Gonzalez et al., 2004; Joubert et al., 2010; Thompson et al., 2002), primary progressive aphasia (Montembeault et al., 2017), or post-stroke aphasia (Vitali et al., 2014). In addition, performance on tests requiring activation of semantic and lexical representations of unique concepts in naming tests (e.g., famous people, famous monuments) appears to be a good predictor of transition from MCI to AD (Ahmed et al., 2008; Estevez-Gonzalez et al., 2004; Thompson et al., 2002).

As already mentioned in the introduction, famous people fluency tasks have not been used very often in studies. They were used in healthy adult participants (Özdemir & Tunçer, 2021; Piolino et al., 2007) as well as in MCI (Gardini et al., 2015) and post-stroke aphasia (Martins & Farrajota, 2007). In a recent study (Macoir et al., 2024), we also showed that individuals with subjective cognitive decline generated significantly fewer famous names in a famous people fluency task compared to their healthy peers. These findings suggest that retrieving the names of famous people in a verbal fluency task may be more challenging for individuals with neurological disorders and may help to objectify cognitive impairment in subjective cognitive decline.

The first aim of this study was to determine the known-group validity, convergent validity, and test–retest validity of the famous people fluency test. The results showed preliminary evidence that the test has good psychometric properties. The results of the test allow the performance of healthy participants to be distinguished from the performance of participants with MCI and participants with AD, while the performance of the latter two groups did not differ significantly. The results further indicated a significant positive correlation between the results of the famous people fluency test and a semantic fluency test, indicating they evaluate a shared cognitive construct (i.e., demonstrating convergent validity). Finally, test–retest analysis showed that the results had good stability over time.

When interpreting cognitive test results, it essential to use normative data that take sociodemographic variables into account and that are culturally and linguistically adapted to the reference population (Mitrushina et al., 2005). This is also particularly important for a new test such as the famous people verbal fluency test described in this study. As far as we know, this study is the first to present normative data for this test. The second objective of this study was to measure the effects of age, education, and sex in a representative sample of French-speaking adults and older people from Quebec in order to obtain normative data for this community. The results of the normative study showed that age and level of education had a significant, albeit small, effect on performance in the famous people verbal fluency test. Therefore, the percentile ranks for performance in the test were stratified according to these two sociodemographic variables.

Although the present study utilized an incidental sampling method, the normative data provided herein stem from a sizable cohort of adults and older adults residing in Quebec. While participants were well distributed across various age groups, it is noteworthy that individuals with higher education levels were overrepresented compared to the general demographics of Quebec. Consequently, caution is advised when interpreting results for individuals with lower levels of education. Moreover, although the normative sample skewed slightly toward more women than men, as sex did not exert a significant impact on performance across the test, the norms appear to be broadly applicable.

The present study had some limitations, and the most important one lies in the characteristics of the normative sample. There were more female than male participants, and this discrepancy was more pronounced among individuals aged 50–69 years. Compared to the provincial demographics, the normative sample also included a higher proportion of individuals with 13 or more years of education compared to individuals with 0–12 years of education. Nonetheless, each age subgroup included a sizable percentage of people with less than 13 years of education, which lends more confidence to the norms for less educated people. The normative sample also included very few people aged 85 and over, and the interpretation of the results in this age group must therefore be made with caution and nuance. Another limitation of the study was that the sample size of participants selected to establish the known-group validity was too small to allow a distinction between participants with MCI and participants with AD. Further studies with larger groups of participants are needed to confirm that the famous people fluency test is suitable for differentiating the performance of people with neurodegenerative diseases.

This study also has significant strengths. First, normative data were obtained based on the performance of a large sample of healthy participants from the French-speaking community in Quebec, Canada. It has been shown that cultural factors influence performance in neuropsychological tests. This effect is evident in non-verbal tasks and even stronger so in tasks involving language, such as verbal fluency tasks (Ardila, 2002; Rosselli & Ardila, 2003). Moreover, to ensure accurate detection of cognitive impairment, it is crucial to establish standards and norms that are appropriate to the cultural context and local practices (Arsenault-Lapierre et al., 2011) and tailored to different sociodemographic factors. For example, educational systems and grading differ across age groups and cultures, which can affect performance on neuropsychological tests. Therefore, future studies are needed to produce normative data adapted to different cultural and linguistic populations. In the present study, the performance of healthy participants on the famous people fluency test was significantly better than that of participants with AD and MCI. Further studies should be conducted to determine the divergent as well as the incremental validity of the test, that is, its additional diagnostic value compared to phonemic and semantic fluency tests. Second, the validity and reliability of the famous people verbal fluency test was supported by data from different groups of participants, increasing confidence in the clinical utility of the assessment tool. Third, this study made a notable contribution to clinical practice by addressing the critical need for culturally specific norms for tests assessing the role of executive functions in language production. As shown in some studies, the famous people verbal fluency test could be useful in various areas of research, including normal and pathological aging. For example, dissociations in the production of common nouns in a classical semantic fluency test and of famous names in the famous people fluency test could provide valuable information about the linguistic, semantic, and executive processes involved in the production of concrete and unique concepts. Finally, the test is short and easy to administer in the clinic. In combination with classical verbal fluency tests, it would therefore be easy to include it in a neuropsychological test battery, and it would provide clinicians with useful information about the integrity or impairment of the linguistic and executive processes involved in the production of the names of unique entities.
